# Cd^2+^ extrusion by P-type Cd^2+^-ATPase of *Staphylococcus aureus* 17810R via energy-dependent Cd^2+^/H^+^ exchange mechanism

**DOI:** 10.1007/s10534-016-9941-5

**Published:** 2016-06-21

**Authors:** Zofia Tynecka, Anna Malm, Zofia Goś-Szcześniak

**Affiliations:** Department of Pharmaceutical Microbiology with Laboratory for Microbiological Diagnostics, Medical University, Chodźki 1, 20-093 Lublin, Poland

**Keywords:** Cd^2+^ resistance, Cd^2+^-ATPase, Energy dependent Cd^2+^/H^+^ exchange, *Staphylococcus aureus*

## Abstract

Cd^2+^ is highly toxic to *Staphylococcus aureus* since it blocks dithiols in cytoplasmic 2-oxoglutarate dehydrogenase complex (ODHC) participating in energy conservation process. However, *S. aureus* 17810R is Cd^2+^-resistant due to possession of *cadA*-coded Cd^2+^ efflux system, recognized here as P-type Cd^2+^-ATPase. This Cd^2+^ pump utilizing cellular energy—ATP, ∆μ_H_^+^ (electrochemical proton potential) and respiratory protons, extrudes Cd^2+^ from cytoplasm to protect dithiols in ODHC, but the mechanism of Cd^2+^ extrusion remains unknown. Here we propose that two Cd^2+^ taken up by strain 17810R via Mn^2+^ uniporter down membrane potential (∆ψ) generated during glutamate oxidation in 100 mM phosphate buffer (high P_i_B) are trapped probably by high affinity sites in cytoplasmic domain of Cd^2+^-ATPase, forming SCdS. This stops Cd^2+^ transport towards dithiols in ODHC, allowing undisturbed NADH production, its oxidation and energy conservation, while ATP could change orientation of SCdS towards facing transmembrane channel. Now, increased number of P_i_-dependent protons pumped electrogenically via respiratory chain and countertransported through the channel down ∆ψ, extrude two trapped cytoplasmic Cd^2+^, which move to low affinity sites, being then extruded into extracellular space via ∆ψ-dependent Cd^2+^/H^+^ exchange. In 1 mM phosphate buffer (low P_i_B), external Cd^2+^ competing with decreased number of P_i_-dependent protons, binds to ψ_s_ of Cd^2+^-ATPase channel, enters cytoplasm through the channel down ∆ψ via Cd^2+^/Cd^2+^ exchange and blocks dithiols in ODHC. However, Mg^2+^ pretreatment preventing external Cd^2+^ countertransport through the channel down ∆ψ, allowed undisturbed NADH production, its oxidation and extrusion of two cytoplasmic Cd^2+^ via Cd^2+^/H^+^ exchange, despite low P_i_B.

## Introduction

Cadmium is highly toxic to living organisms, since it blocks sulphhydryl groups in essential proteins (Vallee and Ulmer 1972; Moulis and Thevenod 2010). Some bacteria carry plasmid-linked *cadA* gene (Novick and Roth 1968; Dyke et al. 1970) conferring Cd^2+^ resistance expressed as decreased ^109^Cd uptake (Chopra 1975; Tynecka et al. 1975; Silver et al. 1982). Tynecka et al. (1981a, 1981b) were the first to report that the highly decreased ^109^Cd uptake by growing cells of Cd^2+^-resistant *Staphylococcus aureus* 17810R was due to pH gradient (∆pH)-dependent, nigericin-sensitive *cadA*-coded Cd^2+^ efflux system.

Subsequently, Silver and coworkers (Nucifora et al. 1989; Silver et al. 1989) showed that the *cadA* gene from staphylococcal plasmid pI258 coded the P-type Cd^2+^-ATPase, belonging to family of membrane-bound, cation-translocating pumps found in eukaryotes and prokaryotes. These pumps located across the membrane maintain homeostasis of essential cations (e.g. Mg^2+^, Ca^2+^, K^+^, Na^+^) or protons (Apell 2003; Kühlbrandt 2004; Pedersen 2007), and confer resistance to heavy metals (e.g. Cd^2+^, Zn^2+^, Cu^2+^) (Rosen 2002; Nies 2003; Kühlbrandt 2004; Silver and Phung 2005; Argüello et al. 2007, 2011). The best characterized is the P-type Ca^2+^-ATPase of sarcoplasmic reticulum (SR) for which detailed biochemical and biophysical data (Apell 2003; Toyoshima 2008) and about 50 crystal structures are available (Toyoshima et al. 2013). However, it is still controversial, how ATP energy is transduced to vectorial Ca^2+^ movement (Scarborough 2003; Toyoshima 2009).

According to sequencing data by Silver and coworkers (Nucifora et al. 1989; Silver et al. 1989), the four cysteine residues present in staphylococcal CadA protein are essential for Cd^2+^-ATPase activity: the conserved Cys23X_2_Cys26 in cytoplasmic domain—a possible high affinity Cd^2+^ binding site, and in conserved Cys371ProCys373 inside transmembrane channel, involved probably in Cd^2+^ extrusion. The CysX_2_Cys motif is related to copper-binding region in Cu^2+^-ATPases (Fan and Rosen 2002) and to mercury-binding region in proteins involved in Hg^2+^ resistance (Barkay et al. 2003). According to Tsai et al. (1992), staphylococcal P-type Cd^2+^-ATPase requires only ATP. Here is shown, that the *cadA*-coded Cd^2+^ efflux system in Cd^2+^-resistant *S. aureus* 17810R (Tynecka et al. Tynecka et al. 1981a, 1981b; Tynecka and Szcześniak 1991) is a P-type Cd^2+^-ATPase requiring: ATP, electrochemical proton potential (∆μ_H_^+^), high phosphate buffer (P_i_B) and P_i_-dependent protons or Mg^2+^. The mechanism of Cd^2+^ extrusion by this staphylococcal Cd^2+^-ATPase is proposed.

## Materials and methods

### Bacterial strains and culture conditions

Cd^2+^-resistant *S.aureus* 17810R, carrying *cadA* gene on penicillinase plasmid pII17810 (Shalita et al. 1980), was described previously (Tynecka et al. 1981a, 1981b). Experiments were performed at 37 °C using early exponential phase cells grown aerobically in 3 % nutrient broth and suspended in 100 mM potassium/sodium phosphate buffer, pH 7 (P_i_B). Cell suspensions were vigorously aerated for 3 h at 37 °C without exogenous electron donor to deprive cells of endogenous energy reserves (Tynecka and Malm 1995; Tynecka et al. 2001). Next, cells were suspended in P_i_B of various concentrations, depending on the experiment, at a density of 0.2 mg dry weight/ml and preincubated with 10 mM glutamate for 10 min at 37 °C (glutamate oxidizing cells). In some experiments, cells were suspended in other buffers: 100 mM triethanolamine/phosphate, pH 7, 100 mM Tris/HCl, pH 7.2 or 100 mM MOPS/NaOH, pH 7. Cd^2+^-sensitive variant strain *S. aureus* 17810S lacking *cadA* gene, also described previously (Tynecka et al. 1981a, 1981b), was used in some experiments as a control organism.

### Reagents

Inhibitors: 2-heptyl-4-hydroxyquinoline N-oxide (HQNO) and dicyclohexylcarbodiimide (DCCD), and ionophores: valinomycin, nigericin or carbonyl cyanide m-chlorophenyl hydrazone (CCCP) were from Sigma (St. Louis, MO). The following radiolabeled compounds were used: ^109^Cd (carrier-free) or sodium [U-^14^C]glutamate (7.4 GBq/mmol)—from Amersham, UK, ^86^RbCl (1.075 GBq/mmol), sodium [^14^C]benzoate (407 MBq/mmol), [^3^H]inulin (3.7 GBq/mmol) or [γ-^32^P]ATP (111 TBq/mmol)—from NEN™ Life Science Products (Boston, MA), while ^32^P_i_—inorganic orthophosphate (740 MBq/mmol)—from the Institute of Nuclear Research, Świerk, Poland.

### Uptake experiments

Uptake of ^109^Cd at 10 μM (as CdCl_2_) by glutamate oxidizing cells of strain 17810R and strain 17810S was assayed by filtration procedure, as described previously (Tynecka et al. 1981a, 1981b). These cells suspended in 100 or 1 mM P_i_B were preincubated at 37 °C for 10 min, with appropriate compounds: MgCl_2_, MnCl_2_ or ionophores—nigericin, valinomycin + KCl or CCCP, depending on the experiment, before addition of 10 μM ^109^CdCl_2_. In order to determine *K*_m_ and *V*_max_ of ^109^Cd uptake in strain 17810R, the initial influx rate of ^109^Cd uptake in 1 mM P_i_B within 1 min at various CdCl_2_ concentrations was measured.

Uptake of ^32^P_i_ (inorganic orthophosphate) by glutamate oxidizing cells of strain 17810R suspended in 100 or 1 mM P_i_B was assayed by filtration procedure, as described previously (Tynecka and Szcześniak 1991).

### Assay of ^109^ Cd efflux

^109^Cd efflux was assayed by filtration procedure, as described previously (Tynecka et al. 1981b). ^109^Cd efflux from washed, glutamate oxidizing cells of strain 17810R was performed after cell preincubation for 20 min with 10 μM ^109^CdCl_2_ in 1 mM P_i_B. After removal of external ^109^CdCl_2_ by cell washing at 4 °C, cells were resuspended in 100 mM P_i_B or in other buffers: 100 mM triethanolamine/phosphate, pH 7, 100 mM Tris/HCl, pH 7.2 or 100 mM MOPS/NaOH, pH 7. DCCD and ionophores: CCCP, valinomycin + KCl or nigericin were added, depending on the experiment. In each experiment 10 mM glutamate was added and all suspensions were prewarmed to 37 °C, before ^109^Cd efflux was measured. ^109^Cd efflux from unwashed, glutamate oxidizing cells was also performed after cell preincubation for 20 min with 10 μM ^109^CdCl_2_ in 1 mM P_i_B. Then, P_i_B concentration was increased at steady-state from 1 mM to 100 mM without cell washing, before ^109^Cd efflux was measured. Appropriate compounds: MgCl_2_ or ionophores: CCCP, valinomycin + KCl or nigericin were added at steady-state, depending on the experiment.

### Assay of ^109^Cd content in subcellular fractions

^109^Cd distribution among subcellular fractions obtained from glutamate oxidizing cells of strain 17810R and strain 17810S preloaded with 10 μM ^109^CdCl_2_ in 100 or 1 mM P_i_B and preincubated at 37 °C with appropriate compounds: MgCl_2_ or ionophores—CCCP, valinomycin + KCl or nigericin, depending on the experiment, was determined according to Tynecka et al. (2001).

### Assay of enzyme activity

Activity of 2-oxoglutarate dehydrogenase complex (ODHC) was measured in the cytoplasmic fraction obtained from glutamate oxidizing cells of strain 17810R suspended in 100 or 1 mM P_i_B and preincubated at 37 °C with CdCl_2_, according to the method described previously (Tynecka and Malm 1996).

### Assay of membrane potential (∆ψ) and pH gradient (∆pH)

The values of ∆ψ and ∆pH in glutamate oxidizing cells of strain 17810R suspended in 100 or 1 mM P_i_B were determined by a filtration procedure from the steady-state distribution of 100 μM ^86^Rb in the presence of 10 μM valinomycin or of 20 μM sodium [^14^C]benzoate, respectively, as described previously (Tynecka et al. 1999). [^3^H]inulin served as a marker for extracellular water.

### Phosphorylation assay

Membrane fraction of strain 17810R was obtained according to the procedure described previously (Tynecka and Malm 1996; Tynecka et al. 2001). Phosphorylation assay was performed as described elsewhere (Tsai and Lynn Linet 1993) with some modifications. To 200 μl of membrane fraction (2.4 mg protein/ml), 2 μl of 1.2 mM EDTA were added, followed by incubation for 10 min, and then 2 μl of 50 μM CdCl_2_ or equivalent volume of deionized water, followed by incubation for 5 min. The reaction was started by addition of 10 μCi of [γ-^32^P]ATP and 2 μl of 0.8 M MgCl_2_. The reaction mixture was incubated at room temperature, then the reaction was stopped after 60 s by addition of equivalent volume of ice-cold 20 % TCA. After 10 min incubation on ice, the membranes were collected by centrifugation (14,000 rpm, 5 min). In order to assay the effect of alkali or hydroxylamine, the pellets were incubated with 100 μl of 0.5 M KOH for 5 min on ice or with 200 μl of 0.1 M sodium acetate containing 260 mM hydroxylamine for 10 min at room temperature. After incubation, equivalent volume of ice-cold 10 % TCA was added. In each case, the collected pellets were washed with water and then twice with 50 mM H_3_PO_4_/NaOH, pH 2.4. Then, the pellets were dissolved in 10 % SDS at 100 °C and suspended in a standard sample buffer used for acidic SDS-PAGE, as described elsewhere (Fairbanks and Avruch 1972). Gels were run at 40 mA for 4–5 h at room temperature. After electrophoresis, autoradiography of the dried gels was performed at 4 °C for 48 h.

### Reproducibility of results

The experimental data shown in each figure are the mean ± SD from at least three independent experiments.

## Results

Highly decreased ^109^Cd accumulation in Cd^2+^-resistant *S. aureus* 17810R oxidizing glutamate in 100 mM phosphate buffer, pH 7 (high P_i_B).

**Fig. 1:**
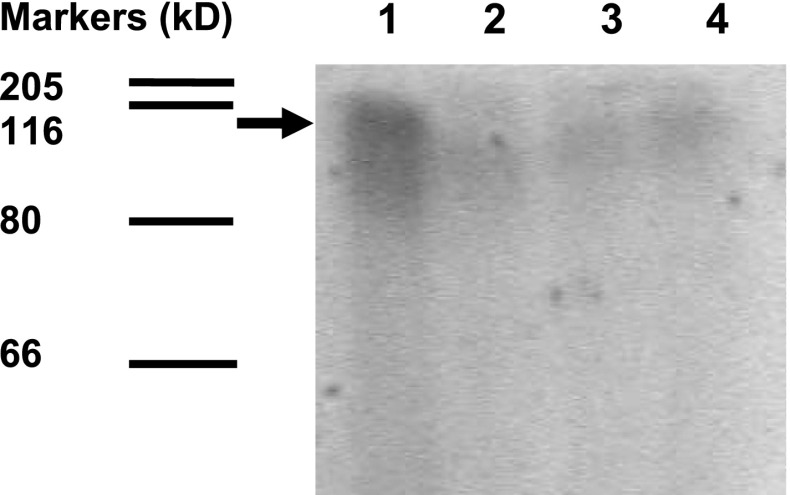
Phosphorylation of membrane proteins in *S. aureus* 17810R by [^32^P]ATP. Lane 1—membrane proteins + 50 μM Cd^2+^, lane 2—membrane proteins + 50 μM Cd^2+^ + 260 mM hydroxylamine, lane 3—membrane proteins + 50 μM Cd^2+^ + 0.5 M KOH, lane 4—membrane proteins without Cd^2+^, the molecular mass markers are also presented. A position of CadA protein is indicated by an arrow

First, membrane proteins of *S. aureus* 17810R harbouring *cadA* gene were phosphorylated by [γ-^32^P]ATP (Fig. 1). The protein band of about 100 kDa was strongly phosphorylated, when Cd^2+^ was present. Intensity of this band was decreased by alkali or hydroxylamine, which is typical for phosphoenzyme intermediate of P-type ATPases (Tsai and Lynn Linet 1993). This suggests that the band strongly phosphorylated in strain 17810R in the presence of Cd^2+^ (Fig. 1) may correspond to CadA protein, having also molecular weight of about 80 kDa (Nucifora et al. 1989; Tsai and Lynn Linet 1993).

**Fig. 2:**
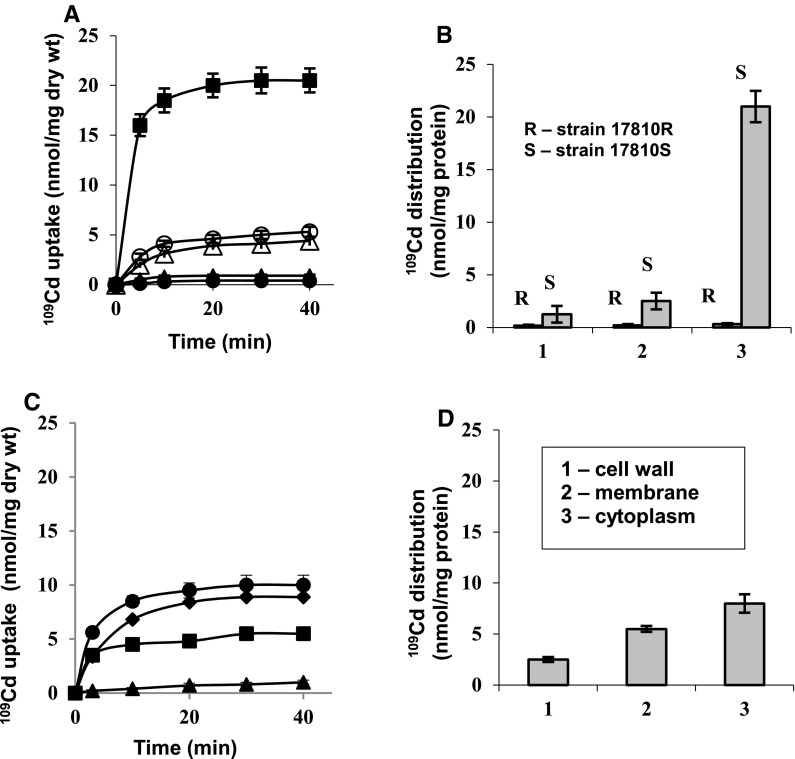
^109^Cd uptake and its distribution in subcellular fractions in *S. aureus* 17810R oxidizing glutamate in 100 mM phosphate buffer, pH 7 (high P_i_B) or in 1 mM phosphate buffer, pH 7 (low P_i_B). In some experiments Cd^2+^-sensitive variant strain *S. aureus* 17810S was used. (**a**) Uptake of ^109^Cd in high P_i_B: control cells of strain 17810R (*filled circles*), cells of strain 17810R preincubated with 0.5 μM nigericin (*filled triangles*), control cells of strain 17810S (*filled squares*), cells of strain 17810S preincubated with 100 μM Mn^2+^ (*empty circles*) or 5 μM valinomycin + 50 mM K^+^ (*empty triangles*). (**b**) Distribution of ^109^Cd in subcellular fractions of strain 17810R and strain 17810S in high P_i_B. (**c**) Uptake of ^109^Cd in low P_i_B: control cells of strain 17810R (*filled circles*), cells of strain 17810R preincubated with 100 μM Mn^2+^ (*filled diamonds*), 1 mM Mg^2+^ (*filled squares*) or 5 mM Mg^2+^ (*filled triangles*). (**d**) Distribution of ^109^Cd in subcellular fractions of strain 17810R in low P_i_B

Cd^2+^-resistant *S. aureus* 17810R took up only 0.5 ± 0.15 nmol ^109^Cd/mg dry wt (Fig. 2a) and accumulated in cytoplasm merely 0.37 ± 0.1 nmol ^109^Cd/mg protein (Fig. 2b). Under similar conditions, the Cd^2+^-sensitive variant strain *S. aureus* 17810S lacking *cadA* gene, took up 20 ± 1.2 nmol ^109^Cd/mg dry wt (Fig. 2a) and accumulated in cytoplasm 21 ± 1.5 nmol ^109^Cd/mg protein (Fig. 2b) down ∆ψ (membrane potential) via high affinity Mn^2+^ uniporter sensitive to Mn^2+^ or valinomycin + K^+^ (Fig. 2a). As was already reported (Tynecka et al. 1981a, 1981b; Tynecka and Malm 1995, 1996: Tynecka et al. 1989), two Cd^2+^ accumulated by strain 17810S in a transport cycle, blocked vicinal dithiols in dihydrolipoate and dihydrolipoate dehydrogenase in the cytoplasmic 2-oxoglutarate dehydrogenase complex (ODHC) in Krebs cycle located in the first energy coupling site of respiratory chain (Tynecka et al. 1999). These dithiols are the only Cd^2+^-sensitive targets in glutamate-linked energy conservation system in strain 17810S; their blocking stopped endogenous NADH production, its oxidation via respiratory chain, generation of electrochemical proton potential (∆μ_H_^+^) and consequently ∆μ_H_^+^-dependent processes without direct blocking of solute transporters and ATP synthase (Tynecka and Malm 1995, 1996; Tynecka et al. 1989, 2001).

The Cd^2+^-resistant strain 17810R did not accumulate Cd^2+^ (Fig. 2a, b), although cells of strain 17810R and 17810S oxidizing glutamate generated ∆μ_H_^+^ of similar value expressed as protonmotive force (∆p) of about −191 ± 5 mV. Data in Fig. 2a, b suggest that two Cd^2+^ transported by strain 17810R via Mn^2+^ uniporter down ∆ψ of −161 ± 5 mV were extruded by Cd^2+^ efflux system described by Tynecka et al. 1981a, 1981b, which was recognized here as a P-type Cd^2+^-ATPase. Cd^2+^ extrusion by this Cd^2+^ pump via Cd^2+^/H^+^ exchange before reaching Cd^2+^-sensitive targets—dithiols in ODHC, allowed undisturbed NADH production (5.4 ± 0.6 nmol NADH/min/mg protein), and consequently its oxidation via respiratory chain, ∆μ_H_^+^ generation and energy conservation (data not shown), rendering host cells Cd^2+^-resistant.

Since nigericin, collapsing ∆pH but stimulating ∆ψ, did not increase Cd^2+^ uptake by strain 17810R (Fig. 2a), this suggested that Cd^2+^ extrusion by Cd^2+^-ATPase from glutamate oxidizing cells was not energized by ΔpH. According to chemiosmotic principles (Mitchell 1966), the enhanced transport of inorganic phosphate (P_i_) by strain 17810R via H^+^/^32^P_i_ symport consuming ΔpH (Tynecka and Szcześniak 1991), could stimulate generation of membrane potential (∆ψ). It is probable that according to Rosenberg and Friedberg (1984) the H^+^/P_i_ cotransport by strain 17810R in high P_i_B could result in phosphate polymerization and accumulation of additional protons in cytoplasm. We suggest that these P_i_-dependent protons pumped electrogenically via respiratory chain could return through the transmembrane channel of Cd^2+^-ATPase down ∆ψ and extruded two cytoplasmic Cd^2+^ into extracellular space via ∆ψ-dependent Cd^2+^/H^+^ exchange. This was confirmed by dependence of Cd^2+^ extrusion on high P_i_B, since below 25 mM P_i_B, linear Cd^2+^ uptake by strain 17810R insensitive to Mn^2+^ was observed (data not shown). To explain the mechanism of Cd^2+^ extrusion by P-type Cd^2+^-ATPase in high P_i_, ^109^Cd uptake by strain 17810R was first characterized in 1 mM P_i_B and then requirements for its net extrusion were studied.

Uptake of ^109^Cd by Cd^2+^-resistant *S. aureus* 17810R oxidizing glutamate in 1 mM phosphate buffer, pH 7 (low P_i_B).

The markedly decreased ^32^P_i_ uptake by strain 17810R from 350 nmol ^32^P_i_/mg dry wt/20 min in high P_i_B to 150 nmol ^32^P_i_/mg dry wt/20 min in low P_i_B could result in decreased number of P_i_-dependent protons pumped electrogenically via respiratory chain. Under these conditions, strain 17810R took up 10 ± 1.3 nmol ^109^Cd/mg dry wt, insensitive to Mn^2+^ (Fig. 2c); about 8 ± 0.9 nmol Cd^2+^/mg protein were found in cell wall and membrane and similar amount of ^109^Cd—in cytoplasm (Fig. 2d), which was only about half of that accumulated by strain 17810S in high P_i_B (Fig. 2b). We suggest that in low P_i_B the external Cd^2+^ could compete with decreased number of P_i_-dependent protons for entry into cytoplasm down ∆ψ through transmembrane channel of Cd^2+^-ATPase. Therefore, the first cytoplasmic Cd^2+^ could be extruded from strain 17810R via exchange with external Cd^2+^ via Cd^2+^/Cd^2+^ exchange, while the second cytoplasmic Cd^2+^ was absent, suggesting its net extrusion. External ^109^Cd uptake in low P_i_B showed linear dependence on Cd^2+^ concentration (data not shown) and high *K*_m_ = 112 ± 2.3 μM and *V*_max_ = 9.1 ± 1.2 nmol Cd^2+^/mg dry wt/min, suggesting that Cd^2+^-ATPase channel may function now as low affinity second pathway transporting external Cd^2+^ down ∆ψ instead of protons towards Cd^2+^–sensitive targets—dithiols in ODHC.

External Cd^2+^ accumulated by strain 17810R in low P_i_B blocked dithiols in ODHC, which stopped NADH production (from 5.4 ± 0.6 to 0.2 ± 0.1 nmol NADH/min/mg protein) and consequently its oxidation via respiratory chain, but ∆μ_H_^+^ generation was unaffected (Δp = −210 ± 4 mV). This suggests that according to Mitchell (1966), Cd^2+^ toxicity to cell respiration could result in conversion of the reversible F_o_F_1_-ATP synthase into hydrolytic direction, which working now as Cd^2+^-insensitive, anaerobic proton pump—F_o_F_1_-ATPase (Tynecka et al. 1990), could continue ∆μ_H_^+^ generation. We suggest that Δψ of −195 ± 4 mV could energize transport of the second cytoplasmic Cd^2+^ via Mn^2+^ uniporter, while ΔpH of 15 ± 2 mV could support its extrusion via Cd^2+^/H^+^ exchange, as confirmed by absence of the second Cd^2+^ in cytoplasm (Fig. 2c, d). Thus, Cd^2+^-ATPase extruded in low P_i_B also two cytoplasmic Cd^2+^, but only external Cd^2+^ reached dithiols in ODHC through the channel via Cd^2+^/Cd^2+^ exchange, disturbing energy conservation and Cd^2+^ resistance of strain 17810R.

We also considered in strain 17810R a controversial problem– existence of low affinity sites on external surface of P-type ATPases (McIntosh 2000; Apell 2003; Scarborough 2003; Toyoshima 2009). First, Silver and coworkers (Nucifora et al. 1989, Silver 1996) recognized during sequencing studies some negatively charged amino acid residues (Glu, Asp) on extracellular surface of CadA protein. It is known (Williams 1978; Barber 1980) that such residues create at physiological pH the surface potentials (ψ_s_) on biological membranes, protected by cations of various protective abilities (Mg^2+^ > Ca^2+^ > K^+^ > Na^+^), depending on their concentration and/or affinity. We suggest that Cd^2+^-ATPase channel in strain 17810R may also possess two negatively charged residues forming surface potential (ψ_s_) functioning as low affinity sites to which protons or external Cd^2+^ may bind before entering the channel, but this depends on P_i_B concentration.

According to Fig. 2c, 1 mM Mg^2+^ prevented external Cd^2+^ uptake by strain 17810R in 50 %, while 5 mM Mg^2+^ (further called Mg^2+^) stopped it. These data confirm existence of low affinity ψ_s_ sites on extracellular surface of Cd^2+^-ATPase channel in strain 17810R. Protection of ψ_s_ by Mg^2+^ against external Cd^2+^ binding and its countertransport through the channel towards dithiols in ODHC allowed undisturbed energy conservation and Cd^2+^ resistance. In contrast, Cd^2+^ uptake by strain 17810S was Mg^2+^-insensitive (data not shown), suggesting ψ_s_ absence in Mn^2+^ uniporter.

**Fig. 3:**
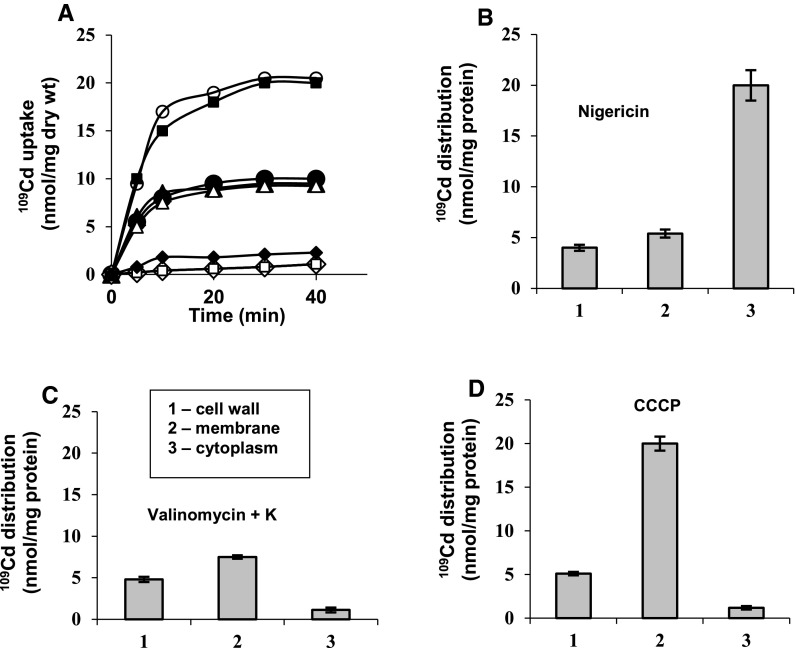
Effects of ionophores on ^109^Cd uptake and its distribution in subcellular fractions in *S. aureus* 17810R oxidizing glutamate in 1 mM phosphate buffer, pH 7 (low P_i_B) with or without 5 mM Mg^2+^. (**a**) ^109^Cd uptake by control cells (*filled circles*) or cells preincubated with 0.5 μM nigericin (*filled squares*), 0.5 μM nigericin + 100 μM Mn^2+^ (*empty triangles*) or 5 mM Mg^2+^ + 0.5 μM nigericin (*filled diamonds*), cells preincubated with 5 μM valinomycin + 50 mM K^+^ (*empty triangles*) or 5 mM Mg^2+^ + 5 μM valinomycin + 50 mM K^+^ (*empty diamonds*), cells preincubated with 10 μM CCCP (*empty circles*) or 5 mM Mg^2+^ + 10 μM CCCP (*empty squares*). Distribution of ^109^Cd in subcellular fractions after cell preincubation with 0.5 μM nigericin (**b**), 5 μM valinomycin + 50 mM K^+^ (**c**) or 10 μM CCCP (**d**)

The ionophore studies in low P_i_B showed that nigericin, collapsing ΔpH, doubled Cd^2+^ uptake by strain 17810R (Fig. 3a). Probably, by stopping ΔpH-dependent efflux of the second cytoplasmic Cd^2+^ energized by the reversed Cd^2+^-insensitive F_o_F_1_-ATPase, nigericin could unmask Δψ-dependent Cd^2+^ transport via Mn^2+^ uniporter, sensitive to Mn^2+^ (Fig. 3a). Now, strain 17810R accumulating in cytoplasm two Cd^2+^ down Δψ—via transmembrane channel (Cd^2+^/Cd^2+^ exchange) and via Mn^2+^ uniporter (Fig. 3a, b), became Cd^2+^-sensitive, like strain 17810S (Fig. 2a, b). However, Mg^2+^ pretreatment of strain 17810R before nigericin addition, prevented external Cd^2+^ binding to ψ_s_ of Cd^2+^-ATPase and also stopped Cd^2+^ countertransport through the channel, rendering host cells Cd^2+^-resistant, despite low P_i_B (Fig. 3a).

Valinomycin + K^+^ collapsing Δψ, did not affect external Cd^2+^ uptake by strain 17810R (Fig. 3a), although Δψ-dependent Cd^2+^ transport via Mn^2+^ uniporter into cytoplasm of strain 17810S was stopped by this ionophore (Fig. 2a). Therefore, valinomycin-insensitive Cd^2+^ uptake by strain 17810R may represent only Δψ-independent external Cd^2+^ binding to cell wall and only to one ψ_s_ site of Cd^2+^-ATPase, prevented by Mg^2+^ (Fig. 3a, c), while external Cd^2+^ binding to the second ψ_s_ site was probably prevented by protons countertransported down unaffected ΔpH.

CCCP also doubled ^109^Cd uptake by strain 17810R (Fig. 3a), although Cd^2+^ accumulation in cytoplasm was stopped (Fig. 3d), since Δψ for Cd^2+^ transport through the channel was blocked by CCCP. This means that two external Cd^2+^ could bind without energy to two ψ_s_ sites in Cd^2+^-ATPase channel (Fig. 3d), prevented by Mg^2+^ (Fig. 3a). These CCCP data strongly confirm existence of two low affinity ψ_s_ sites in Cd^2+^-ATPase channel of strain 17810R.

Restoration of Cd^2+^ resistance in *S. aureus* 17810R by extrusion of Cd^2+^ preaccumulated in 1 mM phosphate buffer, pH 7 (low P_i_B).

Cd^2+^-preloaded cells of strain 17810R in low P_i_ were washed and resuspended in high P_i_B. Since in these Cd^2+^-poisoned cells of strain 17810R the NADH production was blocked and consequently its oxidation, therefore ∆μ_H_^+^ generation and Cd^2+^ extrusion via Cd^2+^/H^+^ exchange were also stopped. Therefore, under such conditions only the Cd^2+^-insensitive proton pump—the reversed F_o_F_1_-ATPase could provide protons for Cd^2+^-ATPase to start Cd^2+^ extrusion from dithiols in ODHC. We suggest that these protons could bind easily to ψ_s_ of Cd^2+^-ATPase channel in washed cells, since there was no extracellular Cd^2+^ to compete. Finally, protons countertransported through the channel down Δψ displaced Cd^2+^ from dithiols in ODHC, which was evidenced by undisturbed ODHC activity (5.6 ± 0.8 nmol NADH/min/mg protein).

**Fig. 4:**
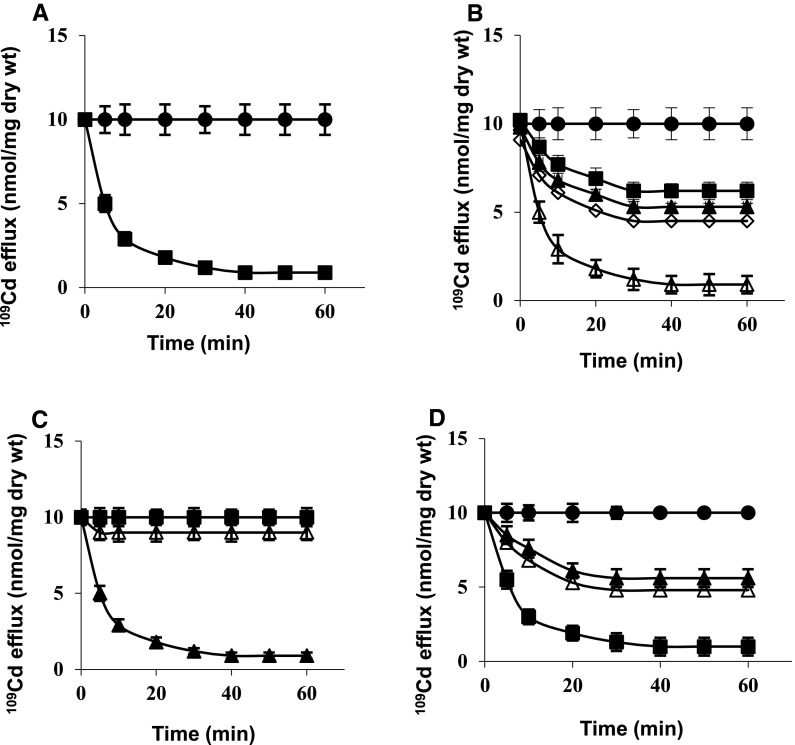
^109^Cd efflux from washed cells of *S. aureus* 17810R preloaded with ^109^Cd in 1 mM phosphate buffer, pH 7 (low P_i_B + glutamate. (**a**) Cd^2+^-preloaded, washed cells were resuspended in 1 mM P_i_B + glutamate (*filled circles*) or in 100 mM P_i_B + glutamate (*filled squares*). (**b**) Cd^2+^-preloaded, washed cells were resuspended in 1 mM P_i_B (*filled circles*) or in 100 mM P_i_B + 10 μM CCCP (*filled squares*), 100 mM P_i_B + 5 μM valinomycin + 50 mM K^+^ (*filled triangles*), 100 mM P_i_B + 100 μM DCCD (*empty diamonds*) or 100 mM 100 mM P_i_B + 0.5 μM nigericin (*empty triangles*), to each suspension glutamate was added. (**c**) Cd^2+^-preloaded, washed cells were resuspended in 100 mM Tris/HCl, pH 7.2 (*filled squares*), 100 mM MOPS/NaOH, pH 7 (*empty triangles*) or in 100 mM triethanolamine/phosphate, pH 7 (*filled triangles*), to each buffer glutamate was added. (**d**) Cd^2+^-preloaded, washed cells were suspended in 1 mM P_i_B (*filled circles*) or in 100 mM triethanolamine/phosphate buffer + 10 μM CCCP (*filled triangles*), 100 mM triethanolamine/phosphate buffer + 5 μM valinomycin + 50 mM K^+^ (*empty triangles*) or 100 mM triethanolamine/phosphate buffer + 0.5 μM nigericin (*filled squares*), to each suspension glutamate was added

Cd^2+^ extrusion was inhibited in 50 % by DCCD, blocking H^+^ channel of F_o_F_1_-ATPase and also by CCCP or valinomycin + K^+^ collapsing Δψ (Fig. 4b). This suggests that Cd^2+^ could be removed from cell wall and ψ_s_ of strain 17810R without energy, but Cd^2+^ extrusion from dithiols in ODHC requiring H^+^ and Δψ, was stopped as evidenced by inhibited ODHC activity with all three compounds (from 1.4 ± 0.2 to 1.5 ± 0.3 nmol NADH/min/mg protein). Only nigericin collapsing ΔpH, allowed Cd^2+^ extrusion down undisturbed Δψ (Fig. 4b). Since CCCP or valinomycin + K^+^ prevented Δψ-dependent proton countertransport through the channel and also stopped Cd^2+^ extrusion, this suggests that dithiols in ODHC (S^–^S^−^) may function in glutamate-linked energy conservation process probably as Δψ generation site.

According to Fig. 4c, other 100 mM buffers containing glutamate-Tris/HCl, pH 7.2 or MOPS/NaOH, pH 7.0, did not initiate Cd^2+^ extrusion from washed cells of strain 17810R. Only 100 mM triethanolamine/phosphate buffer pH 7, triggered total Cd^2+^ efflux (Fig. 4c) sensitive to CCCP or valinomycin + K^+^ in 50 %, but insensitive to nigericin (Fig. 4d). These data strongly confirm requirement of high P_i_ and of P_i_-dependent protons for net Cd^2+^ extrusion by Cd^2+^-ATPase.

**Fig. 5:**
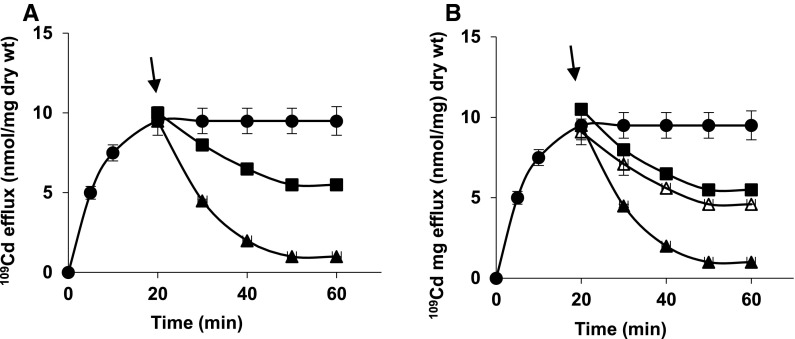
^109^Cd efflux from unwashed cells of *S. aureus* 17810R preloaded with ^109^Cd in 1 mM phosphate buffer, pH 7 (low P_i_B) + glutamate. (**a**) At the time indicated by an arrow, P_i_B concentration was increased from 1 mM to 100 mM (*filled squares*), to one portion of suspension 5 mM Mg^2+^ was added (*filled triangles*), cells suspended in 1 mM P_i_B (*filled circles*). (**b**) At the time indicated by an arrow, P_i_B concentration was increased from 1 mM to 100 mM with the following additions: 5 μM valinomycin + 50 mM K^+^ (*empty triangles*), 10 μM CCCP (*filled squares*) or 0.5 μM nigericin (*filled triangles*), to each suspension 5 mM Mg^2+^ was added; cells suspended in 1 mM P_i_B (*filled circles*)

Cd^2+^ efflux triggered by 100 mM P_i_B from unwashed cells of strain 17810R was incomplete (Fig. 5a), since only Cd^2+^ from cell wall and ψ_s_ could be released without energy, but not Cd^2+^ from cytoplasm requiring protons and Δψ, as evidenced by blocked ODHC activity (1.4 ± 0.3 nmol NADH/min/mg protein). Only high P_i_B plus Mg^2+^—preventing external Cd^2+^ countertransport down Δψ through the channel, allowed Cd^2+^ extrusion from dithiols in ODHC via Cd^2+^/H^+^ exchange (Fig. 5a), as evidenced by unblocked ODHC activity (5.5 ± 0.8 nmol NADH/min/mg protein). This Cd^2+^ efflux was equally affected by ionophores (Fig. 5b), as that from washed cells (Fig. 4b).

## Discussion

**Fig. 6:**
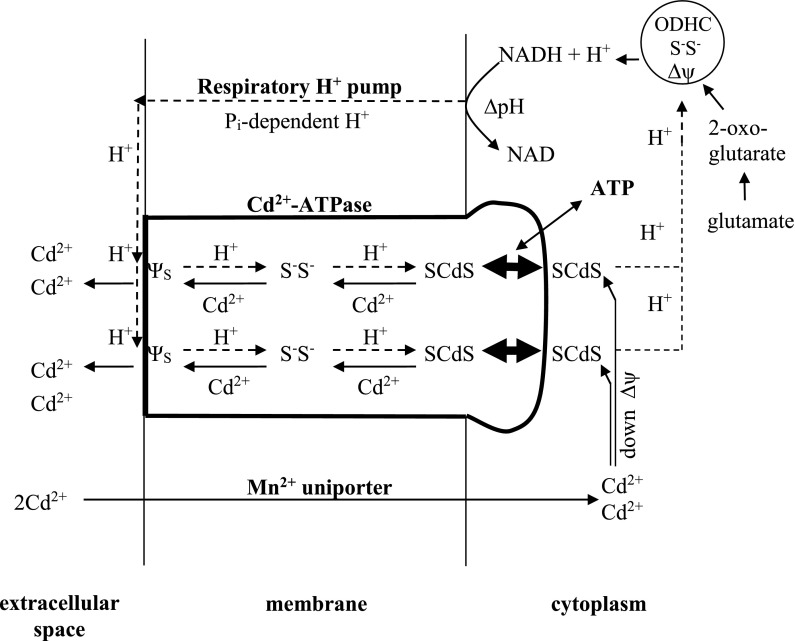
The proposed mechanism of Cd^2+^ extrusion by P-type Cd^2+^-ATPase from *S. aureus* 17810R oxidizing glutamate in 100 mM phosphate buffer, pH 7 (high P_i_B). Two Cd^2+^ transported via Mn^2+^ uniporter down membrane potential (∆ψ) are trapped by high affinity sites—dithiols located in cytoplasmic domain of CadA protein, recognized by Silver and coworkers (Nucifora et al. 1989; Silver et al. 1989). SCdS formation may trigger phosphorylation of CadA protein by ATP, which changes SCdS orientation from facing cytoplasm into facing transmembrane channel. Cd^2+^ trapping stops Cd^2+^ transport towards dithiols in 2-oxoglutarate dehydrogenase complex (ODHC)—the only Cd^2+^-sensitive targets in glutamate-linked energy conservation system, functioning most likely as ∆ψ generation site S^−^S^−^. This allows undisturbed NADH production, its oxidation and ∆μ_H_^+^ generation. Consequently, increased number of P_i_-dependent protons pumped electrogenically via respiratory chain in high P_i_B compete with external Cd^2+^ and bind to low affinity surface sites (ψ_s_) of Cd^2+^-ATPase channel. Finally, protons countertransported down ∆ψ through the channel, extrude two trapped cytoplasmic Cd^2+^ into extracellular space via ∆ψ-dependent Cd^2+^/H^+^ exchange, rendering host cells Cd^2+^-resistant, since the toxic Cd^2+^ could not reach the primary targets—dithiols in ODHC either via Mn^2+^ uniporter or via transmembrane channel

Bacterial Cd^2+^-ATPases belong to the superfamily of P-type ATPases and to the P1 subfamily of soft metal ions pumps (Kühlbrandt 2004). Studies on Cd^2+^-ATPase in *S. aureus* (Nucifora et al. 1989; Silver et al. 1989; Tsai et al. 2002) and in *Listeria monocytogenes* (Bal et al. 2003; Wu et al. 2006a) established amino acid sequence of CadA protein, its membrane topology and suggested involvement in Cd^2+^ extrusion of four cysteine residues present in this protein, but the mechanism of how Cd^2+^ is extruded from Cd^2+^-resistant *S. aureus* remains so far unknown. Also studies on Cd^2+^-ATPase in other microorganisms did not explain this mechanism (Schwager et al. 2012; Schurig-Briccio and Gennis 2012; Chien et al. 2013; Maynaud et al. 2014). As found here, the *cadA*-coded Cd^2+^ efflux system in *S. aureus* 17810R described by Tynecka et al. (1981a, 1981b), appeared to be the P-type Cd^2+^-ATPase. Figure 6 presents a proposed scheme for Cd^2+^ extrusion via Cd^2+^/H^+^ exchange mechanism by the native Cd^2+^-ATPase in *S. aureus* 17810R oxidizing glutamate in high P_i_B.

We propose that two Cd^2+^transported by strain 17810R down Δψ via Mn^2+^ uniporter in high P_i_B are trapped by high affinity sites—dithiols in cytoplasmic domain of staphylococcal Cd^2+^-ATPase, which were recognized by Silver and coworkers (Nucifora et al. 1989; Silver et al. 1989). We suggest that Cd^2+^ trapping (SCdS) stops its transport towards dithiols in cytoplasmic ODHC, allowing undisturbed NADH production, its oxidation via respiratory chain and ∆μ_H_^+^ generation, while ATP could change SCdS orientation from facing cytoplasm towards facing transmembrane channel, probably by a tilting mechanism suggested for Ca^2+^-ATPase (Albers 1967; Post et al. 1972; Higgins and Linton 2001). Finally, increased number of P_i_-dependent protons pumped electrogenically via respiratory chain, could compete with external Cd^2+^ for binding to ψ_s_ of Cd^2+^-ATPase. Then, entering the channel, protons displaced from high affinity sites the two trapped cytoplasmic Cd^2+^, which were transferred through the channel towards low affinity sites ψ_s_, being then extruded into extracellular space via Cd^2+^/H^+^ exchange against electrochemical and concentration gradients (Tynecka et al. 1981a, 1981b), rendering host cells Cd^2+^-resistant. In *Listeria monocytogenes* (Wu et al. 2006b) the *cadA*-coded Cd^2+^-ATPase also extruded two Cd^2+^.

According to the proposed concept (Fig. 6), the P_i_-dependent protons and Cd^2+^ binding ligands in Cd^2+^-ATPase channel of strain 17810R seem to play vital role in Cd^2+^ extrusion. We suggest that the negative charges exposed in the channel by successive proton movement to ψ_s_, CysProCys and CysCys (recognized by Nucifora et al. 1989; Silver et al. 1989; Tsai et al. 2002) can be the driving force for uphill Cd^2+^ pumping through the channel into opposite direction–the extracellular environment. Also Ca^2+^ efflux by Ca^2+^-ATPase from mammalian mitochondria was P_i_-dependent (Roos et al. 1980; Nicholls and Akerman 1982; Ligeti and Lukács 1984). Proton requirement for Cd^2+^ extrusion in strain 17810R, is in accord with Scarbourough`s considerations (Scarborough 2003)—that after phosphorylation reaction, something must weaken ion binding site, allowing Ca^2+^ release into extracellular space. However, the role of protons in SR Ca^2+^-ATPase has been controversial for many years (Ueno and Sekine 1981; Levy et al. 1990; Andersen and Vilsen 1995; Karjalainen et al. 2007; Toyoshima 2009; Fibich and Apell 2011; Bublitz et al. 2013).

Our earlier observations (Tynecka et al. 1981a, 1981b) suggested that protons and external Cd^2+^ could compete for entry into cytoplasm through the channel of Cd^2+^-ATPase down Δψ. Our present data confirm that due to decreased number of P_i_-dependent protons in low P_i_B the external Cd^2+^ could bind to ψ_s_ of Cd^2+^-ATPase channel and then driven down Δψ through the channel via Cd^2+^/Cd^2+^ exchange, blocks dithiols in ODHC, rendering host cells Cd^2+^-sensitive, like strain 17810S.

However, we found that Mg^2+^ can protect strain 17810R against Cd^2+^ poisoning in low P_i_B. According to William’s model (Williams 1978) and our CCCP data, Mg^2+^ can prevent external Cd^2+^ binding to ψ_s_ and stops its countertransport through the channel towards dithiols in ODHC. In energized cells, Mg^2+^ can be displaced transiently by respiratory protons, but still prevents external Cd^2+^ countertransport. Therefore, even a decreased number of P_i_-dependent protons pumped electrogenically during glutamate oxidation in low P_i_B, but protected by Mg^2+^, could enter the channel to extrude two trapped cytoplasmic Cd^2+^ via energy-dependent Cd^2+^/H^+^ exchange. Discharge of ∆μ_H_^+^ by protons allowed Mg^2+^ return to ψ_s_. Such Mg^2+^ oscillation can maintain undisturbed NADH production, its oxidation, energy conservation and Cd^2+^ resistance, despite low P_i_B. Also Ca^2+^ influx and Δψ disruption in mitochondria were prevented by Mg^2+^ (Sharikabad et al. 2001; Racay 2008).

Net Cd^2+^ extrusion requires also steady-state thermodynamic equilibrium between activities of two energy-dependent membrane systems—Mn^2+^ uniporter and Cd^2+^-ATPase. Some changes, e.g. increased Cd^2+^ concentration (Tynecka et al. 1981b), alkaline pH (Tynecka et al. 1981a) or decreased P_i_B concentration shown here, disturb equilibrium and consequently Cd^2+^ resistance. Therefore, the Cd^2+^-ATPase cooperating with P_i_-dependent protons and utilizing cellular energy (ATP and ∆μ_H_^+^) can protect against Cd^2+^ poisoning the vital dithiols in ODHC.

However, we found that Cd^2+^, which blocked dithiols in ODHC in low P_i_B could be also extruded. As was already mentioned, in these Cd^2+^-poisoned cells of strain 17810R, only the Cd^2+^-insensitive, reversed F_o_F_1_-ATPase could pump protons to start the Cd^2+^ efflux process. Besides, we increased the P_i_B concentration to 100 mM (high P_i_B) and also inhibited external Cd^2+^ countertransport down Δψ through the channel either by cell washing or by Mg^2+^ pretreatment. In both situations, the countertransport of P_i_-dependent protons through the channel was restored, leading to Cd^2+^ displacement from dithiols in ODHC. We suggest that the displaced Cd^2+^ could be trapped by high affinity sites of Cd^2+^-ATPase, forming SCdS, while ATP could change SCdS orientation towards facing transmembrane channel. Now, Cd^2+^ displaced by protons from high affinity sites via Cd^2+^/H^+^ exchange moves towards low affinity sites (ψ_s_) of Cd^2+^-ATPase. From here, Cd^2+^ is displaced into extracellular space also via Cd^2+^/H^+^ exchange. Gradual Cd^2+^ extrusion by Cd^2+^-ATPase restored gradually: NADH production, its oxidation, ∆μ_H_^+^ generation via respiratory chain, reversal of F_o_F_1_-ATPase into biosynthetic direction and energy conservation, rendering host cells again Cd^2+^-resistant. DCCD—blocking H^+^ channel of F_o_F_1_-ATPase or CCCP and valinomycin + K^+^ collapsing Δψ, prevented Cd^2+^ extrusion, confirming the requirement of P_i_-dependent protons and of Δψ for the Cd^2+^ efflux process.

To summarize, these studies provide for the first time the novel data on the so far unknown mechanism of Cd^2+^ extrusion by *cadA*-coded P-type Cd^2+^-ATPase in *S. aureus* 17810R, oxidizing glutamate in high P_i_B. Energy-dependent Cd^2+^ extrusion by this pump via Cd^2+^/H^+^ exchange mechanism renders host cells Cd^2+^-resistant, since the toxic Cd^2+^ could not reach the primary Cd^2+^-sensitive targets—dithiols in ODHC via two routes—Mn^2+^ uniporter or transmembrane channel, allowing undisturbed glutamate-linked energy conservation process. Moreover, the vital role of P_i_-dependent protons or Mg^2+^ and of cellular energy (ATP and ∆μ_H_^+^) in Cd^2+^ extrusion by Cd^2+^-ATPase is underlined.
